# Alcohol Consumption Among Adults With a Cancer Diagnosis in the All of Us Research Program

**DOI:** 10.1001/jamanetworkopen.2023.28328

**Published:** 2023-08-10

**Authors:** Mengyao Shi, Chongliang Luo, Oluseye K. Oduyale, Xiaoyu Zong, Noelle K. LoConte, Yin Cao

**Affiliations:** 1 Division of Public Health Sciences, Department of Surgery, Washington University in St Louis School of Medicine, St Louis, Missouri; 2 Division of Hematology, Medical Oncology and Palliative Care, Department of Medicine, University of Wisconsin School of Medicine and Public Health, Madison; 3 University of Wisconsin Carbone Cancer Center, Madison; 4 Alvin J. Siteman Cancer Center, Washington University in St Louis School of Medicine, St Louis, Missouri; 5 Division of Gastroenterology, Department of Medicine, Washington University in St Louis School of Medicine, St Louis, Missouri

## Abstract

**Importance:**

Alcohol consumption is associated with adverse oncologic and treatment outcomes among individuals with a diagnosis of cancer. As a key modifiable behavioral factor, alcohol consumption patterns among cancer survivors, especially during treatment, remain underexplored in the United States.

**Objective:**

To comprehensively characterize alcohol consumption patterns among US cancer survivors.

**Design, Setting, and Participants:**

This cross-sectional study used data from May 6, 2018, to January 1, 2022, from the National Institutes of Health All of Us Research Program, a diverse US cohort with electronic health record (EHR) linkage, and included 15 199 participants who reported a cancer diagnosis and 1839 patients among a subset with EHR data who underwent treatment within the past year of the baseline survey. Data analysis was performed from October 1, 2022, to January 31, 2023.

**Main Outcomes and Measures:**

Prevalence of current drinking and of risky drinking behaviors, including exceeding moderate drinking (>2 drinks on a typical drinking day), binge drinking (≥6 drinks on 1 occasion), and hazardous drinking (Alcohol Use Disorders Identification Test–Consumption [AUDIT-C] score ≥3 for women or ≥4 for men).

**Results:**

This study included 15 199 adults (mean [SD] age at baseline, 63.1 [13.0] years; 9508 women [62.6%]) with a cancer diagnosis. Overall, 11 815 cancer survivors (77.7%) were current drinkers. Among current drinkers, 1541 (13.0%) exceeded moderate drinking, 2812 (23.8%) reported binge drinking, and 4527 (38.3%) engaged in hazardous drinking. After multivariable adjustment, survivors who were younger than 65 years, men, or of Hispanic ethnicity or who received a diagnosis before 18 years of age or ever smoked were more likely to exceed moderate drinking (aged <50 years: odds ratio [OR], 2.90 [95% CI, 2.41-3.48]; aged 50-64 years: OR, 1.84 [95% CI, 1.58-2.15]; men: OR, 2.38 [95% CI, 2.09-2.72]; Hispanic ethnicity: OR, 1.31 [95% CI, 1.04-1.64]; aged <18 years at diagnosis: OR, 1.52 [95% CI, 1.04-2.24]; former smokers: OR, 2.46 [95% CI, 2.16-2.79]; current smokers: OR, 4.14 [95% CI, 3.40-5.04]) or binge drink (aged <50 years: OR, 4.46 [95% CI, 3.85-5.15]; aged 50-64 years: OR, 2.15 [95% CI, 1.90-2.43]; men: OR, 2.10 [95% CI, 1.89-2.34]; Hispanic ethnicity: OR, 1.31 [95% CI, 1.09-1.58]; aged <18 years at diagnosis: OR, 1.71 [95% CI, 1.24-2.35]; former smokers: OR, 1.69 [95% CI, 1.53-1.87]; current smokers: OR, 2.27 [95% CI, 1.91-2.71]). Survivors with cancer diagnosed before 18 years of age or who ever smoked were more likely to be hazardous drinkers (aged <18 years at diagnosis: OR, 1.52 [95% CI, 1.11-2.08]; former smokers: OR, 1.83 [95% CI, 1.68-1.99]; current smokers: OR, 2.13 [95% CI, 1.79-2.53]). Of 1839 survivors receiving treatment as captured in the EHR, 1405 (76.4%) were current drinkers, and among these, 170 (12.1%) exceeded moderate drinking, 329 (23.4%) reported binge drinking, and 540 (38.4%) engaged in hazardous drinking, with similar prevalence across different types of cancer treatment.

**Conclusions and Relevance:**

This cross-sectional study of a diverse US cohort suggests that alcohol consumption and risky drinking behaviors were common among cancer survivors, even among individuals receiving treatment. Given the adverse treatment and oncologic outcomes associated with alcohol consumption, additional research and implementation studies are critical in addressing this emerging concern among cancer survivors.

## Key Points

**Question:**

What is the prevalence of current alcohol consumption and of risky alcohol consumption among cancer survivors in the US?

**Findings:**

In this cross-sectional study of 15 199 adults with a cancer diagnosis from the All of Us Research Program, 77.7% self-reported as current drinkers, and among these, 13.0% exceeded moderate drinking, 23.8% reported binge drinking, and 38.3% engaged in hazardous drinking. Among 1839 survivors receiving cancer treatment, the prevalence of current drinking and risky drinking were similar to the overall cohort and across treatment types.

**Meaning:**

This study suggests that current drinking and risky drinking are common among US cancer survivors even during cancer treatment.

## Teaser

This cross-sectional study uses data from the National Institutes of Health All of Us Research Program to characterize alcohol consumption patterns among US cancer survivors.

## Introduction

With more than 18 million cancer survivors in the United States as of 2022,^1^ identifying modifiable behavioral factors that could improve survivorship and quality of life is a clinical and public health priority. Alcohol consumption, which is ubiquitous in the US and causally linked with multiple types of cancer (oral cavity, pharynx, larynx, esophagus, colorectum, liver, and female breast cancer),^2,3^ is also associated with adverse health outcomes among individuals with a diagnosis of cancer, including higher risks of recurrence^4,5^ or onset of new primary cancers^5,6,7^ as well as death.^4,5,8,9,10,11,12^ In addition, alcohol is associated with worsened treatment outcomes, such as decreased effectiveness and increased risk of complications.^13,14,15,16,17^ Despite these findings, currently, no specific surveillance and counseling guidelines are in place for cancer survivors. Cancer survivors are advised to adhere to the American Cancer Society guideline on nutrition and physical activity for cancer prevention, including (1) that it is best not to drink alcohol and (2) that individuals who choose to drink alcohol should limit alcohol intake to 1 drink or fewer per day for women and 2 drinks or fewer per day for men.^18^

A 2018 statement from the American Society of Clinical Oncology (ASCO) reinforces the need to prioritize alcohol consumption as a key modifiable behavioral factor in the cancer control research agenda.^19^ However, our understanding of alcohol drinking patterns among cancer survivors in the US is limited. Using the National Health Interview Survey (2000-2017), Sanford et al^20^ reported that 35% of cancer survivors who were current drinkers exceeded moderate drinking limits (>1 drink for women and >2 drinks for men) and 21% engaged in binge drinking (≥5 drinks during at least 1 day over the past year). However, to our knowledge, patterns of drinking, including frequency as well as the co-occurrence of multiple risky drinking behaviors, have not been described.^21,22^ The Alcohol Use Disorders Identification Test–Consumption (AUDIT-C) score, a validated score that incorporates frequency of drinking, quantity of drinking, and binge drinking, has been used in primary care and other settings to identify individuals engaging in hazardous drinking.^23,24,25,26^ One study in 17 European countries and Israel reported that 20% of cancer survivors aged 50 years or older engaged in hazardous drinking,^27^ yet such analyses have not been conducted in the US, to our knowledge. More important, although we recently began to recognize the potential adverse effects of drinking during cancer treatment, alcohol consumption patterns during such a critical time window for cancer survivors remain underexplored. To address these knowledge gaps that are critical for short- and long-term survivorship for US cancer survivors, we aimed to comprehensively characterize alcohol consumption patterns among cancer survivors overall and during cancer treatment, using data collected from the All of Us Research Program, a diverse US cohort with electronic health record (EHR) linkage.

## Methods

### Study Population

We identified cancer survivors enrolled in the National Institutes of Health All of Us Research Program, one of the largest, diverse biomedical cohorts within the US.^28,29^ The All of Us Research Program collects data using survey responses, EHR data, biospecimen collection, and physical measurements.^28,30^ The All of Us Research Program institutional review board approved all study procedures. All participants provided written informed consent to share EHRs, surveys, and other study data with qualified investigators for broad-based research. This study followed the Strengthening the Reporting of Observational Studies in Epidemiology (STROBE) reporting guideline.

Among 142 100 participants who completed the Basics, Overall Health, Lifestyle, and Personal Medical History surveys, we identified 15 297 cancer survivors who self-reported a cancer diagnosis (excluding individuals with skin cancer and multiple cancers) from May 6, 2018, to January 1, 2022 (eFigure 1 in Supplement 1). We categorized the cancers as alcohol-related cancers (breast, colon and rectum, and head and neck)^2^ and nonalcohol-related cancers (eTable 1 in Supplement 1). Esophageal cancer was categorized as nonalcohol related because the association with alcohol drinking is confined largely to squamous cell carcinoma,^2^ whereas most cases of esophageal cancer in the US were adenocarcinoma.^31^ Liver cancer was not included in alcohol-related cancers because it was not specifically included in the survey. We also retrieved information on age at cancer diagnosis (child [≤11 years], adolescent [12-17 years], adult [18-64 years], older adult [65-74 years], or elderly adult [≥75 years]) and current treatment status (“Are you currently prescribed medications and/or receiving treatment for this condition?” with an answer of yes or no).

### Assessment of Alcohol Consumption Pattern

Current alcohol consumption status (never, former, and current drinkers) was defined based on the questions in the Lifestyle survey. Participants were asked “In your entire life, have you had at least 1 drink of any kind of alcohol, not counting small tastes or sips?” which was adapted from the National Epidemiologic Survey on Alcohol and Related Conditions. We defined participants who reported not having at least 1 drink of any kind of alcohol as never drinkers, those who had at least 1 drink in their entire life but never had a drink in the past year as former drinkers, and those who had at least 1 drink in the past year as current drinkers. After excluding 98 participants without adequate information to define their current alcohol consumption status, 15 199 cancer survivors were retained in the analyses.

Among current drinkers, we further characterized risky drinking behaviors based on 3 questions: (1) frequency of drinking: “How often did you have a drink containing alcohol in the past year?” with options of never, monthly or less, 2 to 4 times a month, 2 to 3 times a week, or 4 or more times a week; (2) quantity of drinking: “On a typical day when you drink, how many drinks do you have?” with options of 1 or 2, 3 or 4, 5 or 6, 7 to 9, or 10 or more; and (3) binge drinking: “How often did you have 6 or more drinks on 1 occasion in the past year?” with options of never, less than monthly, monthly, weekly, or daily or almost daily. Exceeding moderate drinking was defined from answers about quantity of drinking as participants who drink more than 2 drinks on a typical day when they drink. Binge drinking was defined from the question about binge drinking as participants who ever had 6 or more drinks on 1 occasion. To create the AUDIT-C score (range, 0-12), we added scores of 3 questions with 5 possible answers, which were scored from 0 (less alcohol use) to 4 points (more alcohol use) (eTable 2 in Supplement 1).^24^ Hazardous drinkers included women with AUDIT-C scores of 3 or higher and men with scores of 4 or higher.^24,32,33^

### Assessment of Other Covariates

We included information on age, sex, race and ethnicity, marital status, educational level, annual household income, and insurance status from the Basics survey and general health condition from the Overall Health survey. Sex was categorized based on the question “What was your biological sex assigned at birth?” as women, men, and other sex (including participants who selected “intersex,” “prefer not to answer,” “none of these,” and “skip”). Data on race and ethnicity were collected because prior research has demonstrated different drinking patterns according to racial and ethnic groups.^34,35^ Race and ethnicity were categorized as Hispanic, non-Hispanic Black, non-Hispanic White, and other according to participant self-report. Other race included individuals reporting races other than Hispanic, non-Hispanic Black, or non-Hispanic White (Asian, Middle Eastern or North African, Native Hawaiian or Other Pacific Islander, and participants who responded that none of the provided options fully describe them) and individuals with more than 1 race and ethnicity. Smoking status was assessed in the Lifestyle survey: participants who reported not smoking at least 100 cigarettes in their entire life were categorized as never smokers, those smoking at least 100 cigarettes in their entire life but now do not smoke at all were categorized as former smokers, and those smoking at least 100 cigarettes in the entire life and now smoke every day or some days were categorized as current smokers.

### Ascertainment of Cancer Treatment Using Linked EHR Data

After linking with the EHR,^36^ we identified 10 892 cancer survivors with a first medical encounter 1 year or more before the baseline surveys and a subset of 1839 patients who underwent treatment within the past year of the baseline survey. Treatment was retrieved based on prior studies, using the *Current Procedural Terminology*, 4th Edition; Healthcare Common Procedure Coding System; Systematized Nomenclature of Medicine Clinical Terms; *International Statistical Classification of Diseases and Related Health Problems, Tenth Revision, Procedure Coding System*; and RxNorm.^37,38,39^ We further classified the treatment as surgery, chemotherapy, hormone therapy, radiotherapy, and immunotherapy. We identified treatment modalities that aligned with self-reported cancer type. For surgery, we ensured to include only procedures that matched the specific cancers for which patients received a diagnosis. For instance, we did not count colectomies for any patient without a diagnosis of colorectal cancer.

### Statistical Analysis

Statistical analysis was performed from October 1, 2022, to January 31, 2023. We estimated the crude prevalence of current drinking among cancer survivors as well as the crude prevalence of risky drinking behaviors (including exceeding moderate drinking, binge drinking, and hazardous drinking) among current drinkers. Multivariable logistic regression was used to estimate odds ratios (ORs) and 95% CIs of current drinking and risky drinking behaviors among current drinkers, adjusting for age at survey (<50, 50-64, or ≥65 years), sex (women, men, or other), race and ethnicity (Hispanic, non-Hispanic Black, non-Hispanic White, or other), marital status (never or ever), educational level (<high school, high school or General Educational Development certification, some college, or college), annual household income (<$34 999, $35 000-$74 999, $75 000-$149 999, or ≥$150 000), insurance status (yes or no), smoking status (never, former, or current), cancer type (nonalcohol-related cancers or alcohol-related cancers), age at cancer diagnosis (<18, 18-64, or ≥65 years), and medication and/or receiving treatment (yes or no).

Among the subset of cancer survivors with EHR data who underwent treatment, we estimated the crude prevalence of current drinking and risky drinking behaviors overall and according to type of cancer treatment. To compare with the general population, we conducted secondary analyses to estimate the crude prevalence of current and risky drinking behaviors among survey participants without a prior cancer diagnosis. Data were analyzed in the All of Us Research Workbench (R, version 4.0.2 [R Group For Statistical Computing]).

## Results

In the overall cohort of 15 199 cancer survivors, the mean (SD) age at baseline was 63.1 (13.0) years, 9508 survivors (62.6%) were women, and 11 633 survivors (76.5%) were non-Hispanic White (Table 1). Most cancers (11 515 [75.8%]) were diagnosed when the patient was between 18 and 64 years of age. Most cancer survivors had a college degree (9291 [61.1%]) and a high annual household income, 5333 (35.1%) were former smokers, and 997 (6.6%) were current smokers. Among 1839 cancer survivors who underwent cancer treatment within the past year of the baseline survey, their characteristics were similar to those in the overall cohort (eTable 3 in Supplement 1).

**Table 1.** Characteristics of Participants With Cancer in the All of Us Research Program, According to Sex

Characteristic	Participants, No. (%)			
	Women (n = 9508)	Men (n =5049)	Other (n = 642)^a^	Total (N = 15 199)
Age, mean (SD), y	61.1 (13.1)	66.9 (12.0)	64.4 (13.3)	63.1 (13.0)
Race and ethnicity				
Hispanic	733 (7.7)	274 (5.4)	Removed^b^	1011 (6.7)
Non-Hispanic Black	738 (7.8)	305 (6.0)	Removed^b^	1052 (6.9)
Non-Hispanic White	7402 (77.9)	4144 (82.1)	87 (13.6)	11 633 (76.5)
Other^c^	469 (4.9)	210 (4.2)	Removed^b^	683 (4.5)
Missing	166 (1.7)	116 (2.3)	538 (83.8)	820 (5.4)
Marital status				
Never	1598 (16.8)	626 (12.4)	Removed^b^	2242 (14.8)
Ever	7817 (82.2)	4379 (86.7)	91 (14.2)	12 287 (80.8)
Missing	93 (1.0)	44 (0.9)	533 (83.0)	670 (4.4)
Educational level				
<High school	214 (2.3)	73 (1.4)	Removed^b^	291 (1.9)
High school or GED certification	941 (9.9)	383 (7.6)	Removed^b^	1339 (8.8)
Some college	2526 (26.6)	1071 (21.2)	31 (4.8)	3628 (23.9)
College	5750 (60.5)	3481 (68.9)	60 (9.3)	9291 (61.1)
Missing	77 (0.8)	41 (0.8)	532 (82.9)	650 (4.3)
Annual household income, $				
<35 000	2018 (21.2)	730 (14.5)	34 (5.3)	2782 (18.3)
35 000-75 000	2279 (24.0)	1093 (21.6)	35 (5.5)	3407 (22.4)
75 000-150 000	2542 (26.7)	1597 (31.6)	20 (3.1)	4159 (27.4)
>150 000	1541 (16.2)	1127 (22.3)	Removed^b^	2676 (17.6)
Missing	1128 (11.9)	502 (9.9)	545 (84.9)	2175 (14.3)
Insurance status				
Yes	9289 (97.7)	4941 (97.9)	106 (16.5)	14 336 (94.3)
No	142 (1.5)	68 (1.3)	Removed^b^	212 (1.4)
Missing	77 (0.8)	40 (0.8)	534 (83.2)	651 (4.3)
General health condition				
Excellent	834 (8.8)	515 (10.2)	41 (6.4)	1390 (9.1)
Very good	3131 (32.9)	1840 (36.4)	201 (31.3)	5172 (34.0)
Good	3394 (35.7)	1685 (33.4)	250 (38.9)	5329 (35.1)
Fair	1727 (18.2)	808 (16.0)	120 (18.7)	2655 (17.5)
Poor	362 (3.8)	177 (3.5)	25 (3.9)	564 (3.7)
Missing	60 (0.6)	24 (0.5)	Removed^b^	89 (0.6)
Smoking status				
Never	5595 (58.8)	2648 (52.4)	355 (55.3)	8598 (56.6)
Former	3068 (32.2)	2037 (40.3)	228 (35.5)	5333 (35.1)
Current	676 (7.1)	281 (5.6)	40 (6.2)	997 (6.6)
Missing	169 (1.8)	83 (1.6)	Removed^b^	271 (1.8)
Age at cancer diagnosis, y				
<18	177 (1.9)	88 (1.7)	Removed^b^	284 (1.9)
18-64	7792 (82.0)	3264 (64.6)	459 (71.5)	11 515 (75.8)
≥65	1485 (15.6)	1669 (33.1)	163 (25.4)	3317 (21.8)
Missing	54 (0.6)	28 (0.6)	Removed^b^	83 (0.5)
Medication and/or receiving treatment^d^				
Yes	3460 (36.4)	1836 (36.4)	235 (36.6)	5531 (36.4)
No	5993 (63.0)	3195 (63.3)	404 (62.9)	9592 (63.1)
Missing	55 (0.6)	Removed^b^	Removed^b^	76 (0.5)

Abbreviation: GED, General Educational Development.

^a^ Included participants who selected “intersex,” “prefer not to answer,” “none of these,” and “skip” when they were asked “What was your biological sex assigned at birth?”

^b^ In accordance with All of Us Research Program policy, values corresponding to fewer than 20 participants were removed.

^c^ Included individuals reporting races or ethnicities other than Hispanic, non-Hispanic Black, or non-Hispanic White and individuals with more than 1 race or ethnicity.

^d^ Self-reported current prescription medication and/or treatment in the Personal Medical History survey.

### Current Drinking

**Figure 1.**
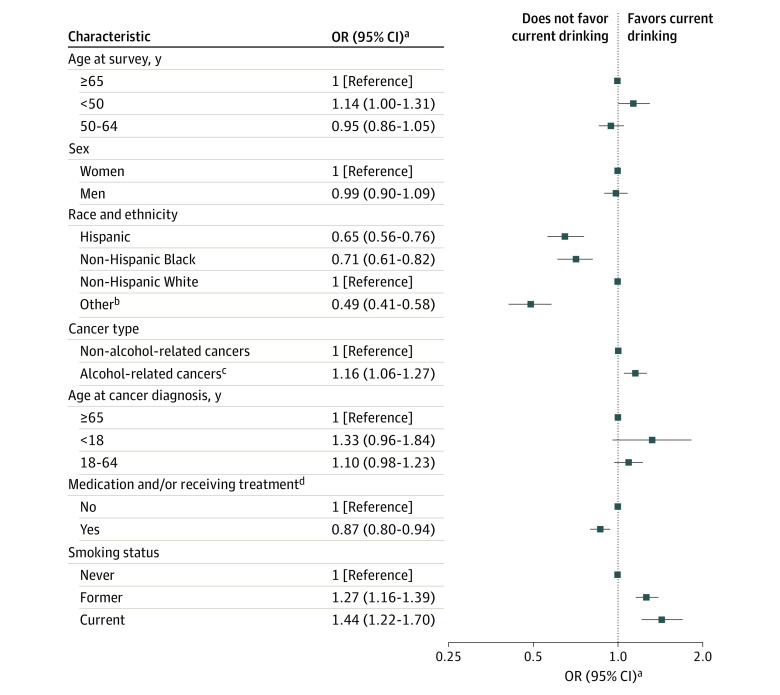
Adjusted Odds Ratios (ORs) of Current Drinking Among Cancer Survivors in the All of Us Research Program ^a^Adjusted for age at survey, sex, race and ethnicity, marital status, educational level, annual household income, insurance status, smoking status, cancer type, age at cancer diagnosis, and currently prescribed medication and/or receiving treatment. ^b^Included individuals reporting races or ethnicities other than Hispanic, non-Hispanic Black, or non-Hispanic White and individuals with more than 1 race or ethnicity. ^c^Included breast, colon and rectum, and head and neck cancer. Esophageal cancer was not included because the association with alcohol drinking is confined largely to squamous cell carcinoma, whereas most cases of esophageal cancer were adenocarcinoma in the US. Liver cancer was not included as it was not specifically included in the All of Us Research Program survey. ^d^Self-reported current medication prescription and/or treatment in the Personal Medical History survey.

Of 15 199 cancer survivors, 11 815 (77.7%) were current drinkers (women, 7344 of 9508 [77.2%]; men, 3971 of 5049 [78.6%]) (Table 2). After multivariable adjustment, survivors who were non-Hispanic White, with alcohol-related cancers, without self-reported current medication prescription and/or treatment, and who were ever smokers were more likely to be current drinkers (Figure 1; eTable 4 in Supplement 1). Compared with non-Hispanic White individuals, survivors who were Hispanic (OR, 0.65; 95% CI, 0.56-0.76), non-Hispanic Black (OR, 0.71; 95% CI, 0.61-0.82), and of other race and ethnicity (OR, 0.49; 95% CI 0.41-0.58) were less likely to be current drinkers. Survivors with alcohol-related cancers were 16% more likely (OR, 1.16; 95% CI, 1.06-1.27) to be current drinkers. Compared with survivors who self-reported they were not currently receiving prescription medication or treatment, those who underwent treatment were less likely to be current drinkers (OR, 0.87; 95% CI, 0.80-0.94). Former smokers (OR, 1.27; 95% CI, 1.16-1.39) and current smokers (OR, 1.44; 95% CI, 1.22-1.70) were also more likely to be current drinkers compared with never smokers.

**Table 2.** Prevalence of Alcohol Consumption Patterns Among Cancer Survivors in the All of Us Research Program, According to Sex

Characteristic	Participants, No. (%)			
	Women (n = 9508)	Men (n = 5049)	Other (n = 642)^a^	Total (N = 15 199)
Alcohol consumption status				
Never	510 (5.4)	176 (3.5)	Removed^b^	704 (4.6)
Former	1654 (17.4)	902 (17.9)	124 (19.3)	2680 (17.6)
Current	7344 (77.2)	3971 (78.6)	500 (77.9)	11 815 (77.7)
Risky drinking behaviors among current drinkers				
Exceeding moderate drinking^c^	777 (10.6)	696 (17.5)	68 (13.6)	1541 (13.0)
Binge drinking^d^				
Any	1560 (21.2)	1119 (28.2)	133 (26.6)	2812 (23.8)
<1 Time/mo	1225 (16.7)	783 (19.7)	100 (20.0)	2108 (17.8)
≥1 Time/mo	335 (4.6)	336 (8.5)	33 (6.6)	704 (6.0)
Hazardous drinking				
AUDIT-C score, mean (SD)^e^	2.51 (1.58)	3.17 (1.90)	2.79 (1.81)	2.74 (1.73)
Hazardous drinking^f^	2946 (40.1)	1581 (39.8)	NA	4527 (38.3)

Abbreviations: AUDIT-C, Alcohol Use Disorders Identification Test–Consumption; NA, not applicable.

^a^ Included participants who selected “intersex,” “prefer not to answer,” “none of these,” and “skip” when asked “What was your biological sex assigned at birth?”

^b^ In accordance with All of Us Research Program policy, values corresponding to fewer than 20 participants were removed.

^c^ Defined as more than 2 drinks on a typical day when drinking in the past year.

^d^ Defined as having 6 or more drinks on 1 occasion in the past year.

^e^ Calculated by adding scores of 3 questions in the AUDIT-C questionnaire.

^f^ Defined as AUDIT-C score of 3 or higher for women and 4 or higher for men in the past year.

### Risky Drinking Behaviors

**Figure 2.**
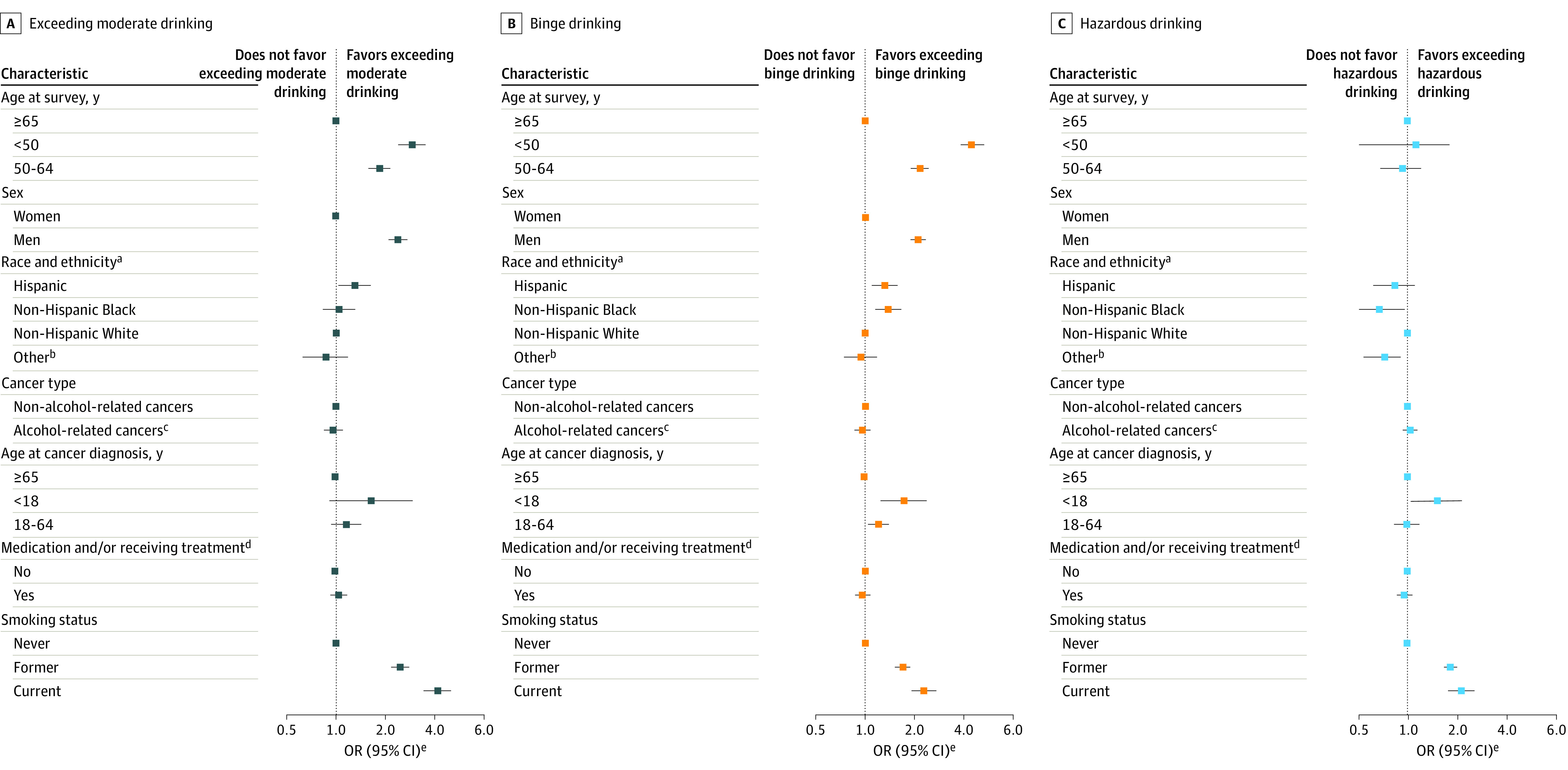
Adjusted Odds Ratios (ORs) of Risky Drinking Behaviors Among Current Drinking Cancer Survivors in the All of Us Research Program ^a^Non-Hispanic White was used as the reference group. ^b^Included individuals reporting races or ethnicities other than Hispanic, non-Hispanic Black, or non-Hispanic White and individuals with more than 1 race or ethnicity. ^c^Included breast, colon and rectum, and head and neck cancer. Esophageal cancer was not included because the association with alcohol drinking is confined largely to squamous cell carcinoma whereas most cases of esophageal cancer were adenocarcinoma in the US. Liver cancer was not included as it was not specifically included in the All of Us Research Program survey. ^d^Self-reported current medication prescription and/or treatment in the Personal Medical History survey. ^e^Adjusted for age at survey, sex, race and ethnicity, marital status, educational level, annual household income, insurance status, smoking status, cancer type, age at cancer diagnosis, and currently prescribed medication and/or receiving treatment.

Of 11 815 survivors who were current drinkers, 1541 (13.0%) exceeded moderate drinking (women, 777 of 7344 [10.6%]; men, 696 of 3971 [17.5%]), and 2812 (23.8%) reported binge drinking (women, 1560 of 7344 [21.2%]; men, 1119 of 3971 [28.2%]) (Table 2; eFigure 2 in Supplement 1). After multivariable adjustment, survivors who were younger than 65 years, who were men, who were Hispanic, with cancer diagnosed before 18 years of age, or who ever smoked were more likely to exceed moderate drinking (aged <50 years: odds ratio [OR], 2.90 [95% CI, 2.41-3.48]; aged 50-64 years: OR, 1.84 [95% CI, 1.58-2.15]; men: OR, 2.38 [95% CI, 2.09-2.72]; Hispanic ethnicity: OR, 1.31 [95% CI, 1.04-1.64]; aged <18 years at diagnosis: OR, 1.52 [95% CI, 1.04-2.24]; former smokers: OR, 2.46 [95% CI, 2.16-2.79]; current smokers: OR, 4.14 [95% CI, 3.40-5.04]) and engage in binge drinking (aged <50 years: OR, 4.46 [95% CI, 3.85-5.15]; aged 50-64 years: OR, 2.15 [95% CI, 1.90-2.43]; men: OR, 2.10 [95% CI, 1.89-2.34]; Hispanic ethnicity: OR, 1.31 [95% CI, 1.09-1.58]; aged <18 years at diagnosis: OR, 1.71 [95% CI, 1.24-2.35]; former smokers: OR, 1.69 [95% CI, 1.53-1.87]; current smokers: OR, 2.27 [95% CI, 1.91-2.71]) (Figure 2; eTable 5 in Supplement 1). The odds of engaging in more than moderate drinking or binge drinking were similar among current drinkers who reported receiving medication and/or undergoing treatment and those who did not.

A total of 4527 current drinkers (38.3%) engaged in hazardous drinking, defined by an AUDIT-C score of 3 or higher for women and 4 or higher for men, with similar prevalences among women and men. After multivariable adjustment, survivors with cancer diagnosed before 18 years of age were more likely to be hazardous drinkers (OR, 1.52; 95% CI, 1.11-2.08) compared with those diagnosed at 65 years of age or older (eTable 5 in Supplement 1). Compared with never smokers, former smokers were 83% more likely (OR, 1.83; 95% CI, 1.68-1.99) to be hazardous drinkers, and current smokers had more than 2-fold the odds (OR, 2.13; 95% CI, 1.79-2.53) of engaging in hazardous drinking. For survivors with the highest risk of hazardous drinking (current smokers who received a cancer diagnosis before 18 years of age), their risky drinking behaviors were associated with more frequent, heavy drinking as well as binge drinking (eFigure 3 in Supplement 1). No association was observed between self-reported receipt of medication or treatment and hazardous drinking. Of 119 977 survey participants without a prior cancer diagnosis, 96 058 (80.1%) were current drinkers; among these, 19 949 (20.8%) exceeded moderate drinking, 34 135 (35.5%) reported binge drinking, and 48 090 (50.1%) engaged in hazardous drinking (eTable 6 in Supplement 1).

### Alcohol Consumption Patterns Among a Subset With Past-Year Treatment

Of 1839 cancer survivors who received treatment within the past year of the baseline survey, 1405 (76.4%) self-reported as current drinkers (Table 3), similar to the prevalence in the overall cohort of patients who self-reported receiving medication and/or treatment and being current drinkers (4211 of 5531 [76.1%]). This prevalence was largely similar for each cancer treatment, with the highest for patients who underwent surgery (329 of 409 [80.4%]) (Table 3). Of 1405 current drinkers who received treatment within the past year of the baseline survey, 170 (12.1%) exceeded moderate drinking, 329 (23.4%) reported binge drinking, and 540 (38.4%) engaged in hazardous drinking.

**Table 3.** Prevalence of Alcohol Consumption Patterns Among Cancer Survivors in the All of Us Research Program, According to Cancer Treatment During the Same Year^a^

Characteristic	Participants, No. (%)					
	Surgery (n = 409)	Chemotherapy (n = 481)	Hormone therapy (n = 1001)	Radiotherapy (n = 224)	Immunotherapy (n = 295)	Total (N = 1839)
Alcohol consumption status						
Never	Removed^b^	32 (6.7)	40 (4.0)	Removed^b^	20 (6.8)	82 (4.5)
Former	63 (15.4)	107 (22.2)	169 (16.9)	43 (19.2)	72 (24.4)	352 (19.1)
Current	329 (80.4)	342 (71.1)	792 (79.1)	172 (76.8)	203 (68.8)	1405 (76.4)
Risky drinking behaviors among current drinkers						
Exceeding moderate drinking^c^	49 (14.9)	46 (13.5)	80 (10.1)	23 (13.4)	Removed^b^	170 (12.1)
Binge drinking^d^						
Any	93 (28.3)	77 (22.5)	180 (22.7)	36 (20.9)	36 (17.7)	329 (23.4)
<1 Time/mo	70 (21.3)	56 (16.4)	149 (18.8)	26 (15.1)	28 (13.8)	262 (18.6)
≥1 Time/mo	23 (7.0)	21 (6.2)	31 (3.9)	Removed^b^	Removed^b^	67 (4.8)
Hazardous drinking						
AUDIT-C score, mean (SD)^e^	2.79 (1.70)	2.54 (1.66)	2.54 (1.52)	2.68 (1.84)	2.44 (1.39)	2.61 (1.58)
Hazardous drinking^f^	135 (41.0)	117 (34.2)	316 (39.9)	64 (37.2)	69 (34.0)	540 (38.4)

Abbreviation: AUDIT-C, Alcohol Use Disorders Identification Test–Consumption.

^a^ After restricting to patients with electronic health record data 1 year or more before the baseline survey, we identified 1839 patients with any type of the listed cancer treatment within 1 year of the baseline of survey.

^b^ In accordance with All of Us Research Program policy, values corresponding to fewer than 20 participants were removed.

^c^ Defined as more than 2 drinks on a typical day when drinking in the past year.

^d^ Defined as having 6 or more drinks on 1 occasion in the past year.

^e^ Calculated by adding scores of 3 questions in the AUDIT-C questionnaire.

^f^ Defined as AUDIT-C score of 3 or higher for women and 4 or higher for men in the past year.

## Discussion

Our study extends the scope of prior understanding through using a diverse US cohort to characterize risky drinking behaviors comprehensively among cancer survivors. We again highlight that alcohol consumption and risky drinking behaviors are common among cancer survivors, and we found that, among current drinkers, men, Hispanic individuals, those with cancer diagnosed before 18 years of age, and smokers are more likely to engage in risky drinking behaviors. More important, by linking with EHR data to annotate treatment information, we found that drinking and risky drinking behaviors are prevalent even among individuals concurrently receiving treatment for cancer.

Similar to a prior study using a nationally representative survey,^20^ we found that most cancer survivors were current drinkers, and non-Hispanic White individuals or ever smokers were more likely to be current drinkers. In addition, we found that survivors with alcohol-related cancers or without self-reported current treatment were more likely to be current drinkers. Also in line with the previous study,^20^ we found that, among current drinkers, survivors who were younger, men, Hispanic, and ever smokers were more likely to exceed moderate drinking or binge drink. Comparable with previous findings,^40^ our study also suggested that Hispanic individuals are less likely to drink compared with non-Hispanic White individuals, but Hispanic individuals who choose to drink are more likely to consume higher volumes of alcohol, possibly due in part to acculturation.^41^ Although adolescent or young adult cancer survivors were reported to be more likely than peers without cancer to drink alcohol,^42^ our study found that survivors with cancer diagnosed before 18 years of age were more likely to engage in both heavy and binge drinking. Using validated AUDIT-C scores that incorporate frequency of drinking, quantity of drinking, and binge drinking, we reported for the first time, to our knowledge, that 38.3% of cancer survivors in this diverse US cohort engaged in hazardous drinking. This higher prevalence compared with those reported in Europe by Bosque-Prous et al^27^ might be explained in part by using lower cutoff points to define hazardous drinking in our study (AUDIT-C scores of ≥3 for women and ≥4 for men) vs those used by Bosque-Prous et al^27^ (AUDIT-C scores of ≥4 for women and ≥5 for men). Although more studies are warranted, the high prevalence of cancer survivors engaged in hazardous drinking highlights the need for immediate interventions to reduce alcohol intake among US cancer survivors.

Alcohol consumption and risky drinking behaviors among cancer survivors are associated with various adverse long-term outcomes, including higher risk of recurrence,^4,5^ secondary primary tumors,^5,6,7^ and increased mortality.^4,5,8,9,10,11,12^ In a meta-analysis involving 209 597 cancer survivors, alcohol consumption was associated with a 17% increased risk of cancer recurrence and an 8% increased risk of overall mortality.^4^ More studies are warranted to elucidate the role of each risky drinking behavior and the overall pattern in long-term outcomes. Survivors with cancer diagnosed before 18 years of age or ever smokers were more likely to be hazardous drinkers. Because of the persistent excess risks for second primary cancers throughout the life course for childhood cancer survivors^43,44,45^ and the elevated risks for alcohol- and tobacco-related secondary primary cancers among drinkers who ever smoke,^6^ targeted efforts for alcohol reduction are needed for these 2 groups of survivors who are more susceptible.

As highlighted in the 2018 ASCO statement,^19^ in addition to long-term survivorship, accumulating data support the associations between alcohol drinking and treatment outcomes among cancer survivors. For instance, alcohol use worsens postsurgical outcomes, including increased risk of surgical complications, longer hospitalizations, more surgical procedures, prolonged recovery, higher health care costs,^46,47,48^ and higher mortality.^19,49^ Alcohol use during and after radiotherapy is associated with a higher risk of osteonecrosis of the jaw among patients with head and neck cancers.^50,51,52,53^ In addition, alcohol is well known to have neurotoxic, cardiotoxic, and hepatotoxic effects.^54,55,56^ Among patients undergoing chemotherapy, alcohol has been suggested to worsen cognition and cardiotoxicity.^57,58^ Furthermore, alcohol use is associated with hepatic dysfunction and regulates cytochrome enzymatic activity,^54^ which is important for the metabolism of chemotherapeutic agents and possibly alters their effectiveness or toxic effects. Although the association of alcohol use with immunotherapy for cancer is unclear, the treatment outcomes may be somewhat affected due to alcohol-induced immune dysfunction.^59^

Our understanding of alcohol consumption patterns among cancer survivors receiving treatment has just begun to emerge. In a recent pilot study of 69 patients in Wisconsin, 30% of cancer survivors reported drinking alcohol while receiving chemotherapy, and 38% of these drinkers reported at least some complications.^60^ To date, the All of Us Research Program is the only national cohort that allows us to capture alcohol consumption patterns in the context of cancer treatment. Unexpectedly, a large proportion of cancer survivors undergoing cancer treatment were current drinkers (76.4%) or were engaged in risky drinking (exceeding moderate drinking, 12.1%; binge drinking, 23.4%; hazardous drinking, 38.4%); these proportions were similar across different types of cancer treatment as well as in the overall cohort. Taken together, our findings point to the immediate and unmet need to intervene on the behalf of individuals with risky drinking behaviors in oncologic care settings. Clinicians should collect alcohol consumption information while also informing survivors of the potential harms in an effort to reduce risky alcohol use. Given that drinking is deeply ingrained in societal norms and rituals, and considering the limited awareness of how alcohol consumption is associated with cancer outcomes, it is imperative to provide support to patients who are identified as alcohol users and offer them guidance. Our findings also call for large-scale epidemiologic studies to further evaluate the association of alcohol with therapeutic efficacy and treatment outcomes among cancer survivors.

### Strengths and Limitations

This study has some strengths, including the use of a large and diverse national cohort to comprehensively characterize risky drinking behaviors, including hazardous drinking, whereas previous studies focused on exceeding moderate drinking and binge drinking only. More important, we used the EHR linkages to retrieve information on cancer treatment.

Our study also has several limitations. First, per the Dietary Guidelines for Americans 2020-2025, exceeding moderate drinking was defined as having more than 1 drink per day for women.^61^ However, the All of Us Research Program survey only allowed us to define exceeding moderate drinking among women as having more than 2 drinks. Similarly, we characterized patients who consumed 6 or more drinks on 1 occasion as binge drinkers, instead of those who consumed 4 or more drinks for women or 5 or more drinks for men per the National Institute on Alcohol Abuse and Alcoholism guideline.^62^ However, with these underestimates, the prevalence of women exceeding moderate drinking was high, as was the prevalence of binge drinking among both women and men, which further highlight the pressing need for reduction of alcohol consumption. Second, because the All of Us Research Program survey asked about average alcohol consumption in the past year, we retrieved cancer treatment information during the same time in the EHR. However, the exact timing of alcohol consumption in association with cancer treatment was not clear. Additional studies are required to validate and refine our findings.

## Conclusions

This cross-sectional study found that current and risky drinking (exceeding moderate drinking, binge drinking, and hazardous drinking) were common among US cancer survivors even during cancer treatment. Given the short- and long-term adverse treatment and oncologic outcomes associated with alcohol consumption, additional research and implementation studies are critical to address this emerging concern among cancer survivors.

## Notes

Supplement 1. **eTable 1.** Cancer Characteristics According to Sex, *All of Us* Research Program **eTable 2.** Alcohol Use Disorders Identification Test–Consumption (AUDIT-C) **eTable 3.** Characteristics of Cancer Survivors Who Underwent Cancer Treatment Within 1 Year Before the Baseline Survey, *All of Us* Research Program **eTable 4.** Adjusted Odds Ratios of Current Drinking Among Cancer Survivors, *All of Us* Research Program **eTable 5.** Adjusted Odds Ratios of Risky Drinking Behaviors Among Current Drinking Cancer Survivors, *All of Us* Research Program **eTable 6.** Prevalence of Alcohol Consumption Patterns Among Survey Participants Without Prior Cancer Diagnosis According to Sex, *All of Us* Research Program **eFigure 1.** Flow Chart of the Study Population **eFigure 2.** (A) Mean AUDIT-C Score Among Cancer Survivors According to Sex; (B) Venn Diagram Showing Cancer Survivors Engaged in Exceeding Moderate Drinking, Binge Drinking and Hazardous Drinking Among 11815 Current Drinkers, *All of Us* Research Program **eFigure 3.** Mean AUDIT-C Score Among Cancer Survivors According to Age at Cancer Diagnosis and Smoking Status, *All of Us* Research Program

Supplement 2.
Data Sharing Statement
