# Supplementary material for: Alcohol Consumption Among Adults With a Cancer Diagnosis in the All of Us Research Program
Source: JAMA Netw Open. 2023 Aug 10;6(8):e2328328. doi: 10.1001/jamanetworkopen.2023.28328 (PMC10415957; doi:10.1001/jamanetworkopen.2023.28328)
Supplement: Supplement 1. — eTable 1. Cancer Characteristics According to Sex, All of Us Research Program eTable 2. Alcohol Use Disorders Identification Test–Consumption (AUDIT-C) eTable 3. Characteristics of Cancer Survivors Who Underwent Cancer Treatment Within 1 Year Before the Baseline Survey, All of Us Research Program eTable 4. Adjusted Odds Ratios of Current Drinking Among Cancer Survivors, All of Us Research Program eTable 5. Adjusted Odds Ratios of Risky Drinking Behaviors Among Current Drinking Cancer Survivors, All of Us Research Program eTable 6. Prevalence of Alcohol Consumption Patterns Among Survey Participants Without Prior Cancer Diagnosis According to Sex, All of Us Research Program eFigure 1. Flow Chart of the Study Population eFigure 2. (A) Mean AUDIT-C Score Among Cancer Survivors According to Sex; (B) Venn Diagram Showing Cancer Survivors Engaged in Exceeding Moderate Drinking, Binge Drinking and Hazardous Drinking Among 11815 Current Drinkers, All of Us Research Program eFigure 3. Mean AUDIT-C Score Among Cancer Survivors According to Age at Cancer Diagnosis and Smoking Status, All of Us Research Program [file jamanetwopen-e2328328-s001.pdf]

Supplementary Online Content

Shi M, Luo C, Oduyale OK, Zong X, LoConte NK, Cao Y. Alcohol consumption among adults with a cancer diagnosis in the All of Us Research Program. *JAMA Netw Open.* 2023;6(8):e2328328. doi:10.1001/jamanetworkopen.2023.28328

eTable 1. Cancer Characteristics According to Sex, *All of Us* Research Program
eTable 2. Alcohol Use Disorders Identification Test–Consumption (AUDIT-C)
eTable 3. Characteristics of Cancer Survivors Who Underwent Cancer Treatment Within 1 Year Before the Baseline Survey, *All of Us* Research Program
eTable 4. Adjusted Odds Ratios of Current Drinking Among Cancer Survivors, *All of Us* Research Program
eTable 5. Adjusted Odds Ratios of Risky Drinking Behaviors Among Current Drinking Cancer Survivors, *All of Us* Research Program
eTable 6. Prevalence of Alcohol Consumption Patterns Among Survey Participants Without Prior Cancer Diagnosis According to Sex, *All of Us* Research Program
eFigure 1. Flow Chart of the Study Population
eFigure 2. (A) Mean AUDIT-C Score Among Cancer Survivors According to Sex; (B) Venn Diagram Showing Cancer Survivors Engaged in Exceeding Moderate Drinking, Binge Drinking and Hazardous Drinking Among 11815 Current Drinkers, *All of Us* Research Program
eFigure 3. Mean AUDIT-C Score Among Cancer Survivors According to Age at Cancer Diagnosis and Smoking Status, *All of Us* Research Program

This supplementary material has been provided by the authors to give readers additional information about their work.

© 2023 Shi M et al. *JAMA Network Open.*

eTable 1. Cancer Characteristics According to Sex, *All of Us* Research Program

Characteristics	Women	Men	Other[a]	Total
**No. of participants**	9508	5049	642	15199
**Cancer type**				
Alcohol-related cancers[b]				
Breast	4247 (44.7%)	27 (0.5%)	168 (26.2%)	4442 (29.2%)
Colon & rectum	362 (3.8%)	318 (6.3%)	29 (4.5%)	709 (4.7%)
Head & neck	98 (1.0%)	166 (3.3%)	~	276 (1.8%)
Non-alcohol-related cancers				
Bladder	104 (1.1%)	287 (5.7%)	~	409 (2.7%)
Blood	561 (5.9%)	515 (10.2%)	50 (7.8%)	1126 (7.4%)
Bone	59 (0.6%)	89 (1.8%)	~	154 (1.0%)
Brain	88 (0.9%)	91 (1.8%)	~	182 (1.2%)
Cervical	980 (10.3%)	~	41 (6.4%)	1021 (6.7%)
Endocrine	52 (0.5%)	45 (0.9%)	~	101 (0.7%)
Endometrial	372 (3.9%)	~	~	386 (2.5%)
Esophagus	~	71 (1.4%)	~	92 (0.6%)
Eye	27 (0.3%)	20 (0.4%)	~	49 (0.3%)
Kidney	209 (2.2%)	223 (4.4%)	~	449 (3.0%)
Lung	241 (2.5%)	132 (2.6%)	20 (3.1%)	393 (2.6%)
Other Site	1006 (10.6%)	772 (15.3%)	74 (11.5%)	1852 (12.2%)
Ovarian	304 (3.2%)	~	~	315 (2.1%)
Pancreatic	46 (0.5%)	61 (1.2%)	~	109 (0.7%)
Prostate	~	2061 (40.8%)	124 (19.3%)	2185 (14.4%)
Stomach	37 (0.4%)	~	~	57 (0.4%)
Thyroid	699 (7.4%)	155 (3.1%)	38 (5.9%)	892 (5.9%)

a. Other included participants who selected ‘intersex’, ‘prefer not to answer’, ‘none of these’, and ‘skip’ when asked ‘What was your biological sex assigned at birth?’.
b. Esophageal cancer was not included since the association with alcohol drinking is largely confined to squamous cell carcinoma whereas the majority esophageal cancer were adenocarcinoma in the US. Liver cancer was not included as it was not specifically asked in the *All of Us* Research Program survey.
In accordance with *All of Us* Research Program policy, values corresponding to fewer than 20 participants were removed from the table (labeled as ~).

© 2023 Shi M et al. *JAMA Network Open.*

eTable 2. Alcohol Use Disorders Identification Test–Consumption (AUDIT-C)

	Points
**Q1: How often did you have a drink containing alcohol in the past year?**	
Never	0
Monthly or less	1
Two to four times a month	2
Two to three times a week	3
Four or more times a week	4
**Q2: On a typical day when you drink, how many drinks did you have?**	
1 or 2	0
3 or 4	1
5 or 6	2
7 to 9	3
10 or more	4
**Q3: How often did you have six or more drinks on one occasion in the past year?**	
Never	0
Less than monthly	1
Monthly	2
Weekly	3
Daily or almost daily	4

© 2023 Shi M et al. *JAMA Network Open.*

eTable 3. Characteristics of Cancer Survivors Who Underwent Cancer Treatment Within 1 Year Before the Baseline Survey, *All of Us* Research Program[a]

Characteristics	Women	Men	Other[b]	Total
**No. of participants**	1324	454	61	1839
**Age**				
Mean (SD)	59.9 (12.4)	65.6 (11.4)	63.3 (11.3)	61.4 (12.3)
**Race/ethnicity**				
Hispanic	93 (7.0%)	23 (5.1%)	~	117 (6.4%)
Non-Hispanic Black	100 (7.6%)	34 (7.5%)	~	136 (7.4%)
Non-Hispanic White	1058 (79.9%)	371 (81.7%)	~	1440 (78.3%)
Other[c]	49 (3.7%)	~	~	68 (3.7%)
Missing	24 (1.8%)	~	46 (75.4%)	78 (4.2%)
**Marital status**				
Never	208 (15.7%)	65 (14.3%)	~	277 (15.1%)
Ever	1103 (83.3%)	388 (85.5%)	~	1502 (81.7%)
Missing	~	~	46 (75.4%)	60 (3.3%)
**Education level**				
<High School	21 (1.6%)	~	~	32 (1.7%)
High School/GED	116 (8.8%)	36 (7.9%)	~	155 (8.4%)
Some college	351 (26.5%)	100 (22.0%)	~	455 (24.7%)
College	832 (62.8%)	305 (67.2%)	~	1144 (62.2%)
Missing	~	~	46 (75.4%)	53 (2.9%)
**Annual household income**				
<35k	234 (17.7%)	64 (14.1%)	~	305 (16.6%)
35-75k	274 (20.7%)	78 (17.2%)	~	354 (19.2%)
75-150k	393 (29.7%)	140 (30.8%)	~	538 (29.3%)
>150k	263 (19.9%)	113 (24.9%)	~	377 (20.5%)
Missing	160 (12.1%)	59 (13.0%)	46 (75.4%)	265 (14.4%)
**Insurance status**				
Yes	1307 (98.7%)	441 (97.1%)	~	1763 (95.9%)
No	~	~	~	~
Missing	~	~	46 (75.4%)	60 (3.3%)
**General health condition**				
Excellent	87 (6.6%)	24 (5.3%)	~	114 (6.2%)
Very Good	420 (31.7%)	150 (33.0%)	~	583 (31.7%)
Good	504 (38.1%)	159 (35.0%)	29 (47.5%)	692 (37.6%)
Fair	249 (18.8%)	95 (20.9%)	~	357 (19.4%)
Poor	61 (4.6%)	23 (5.1%)	~	87 (4.7%)
Missing	~	~	~	~
**Smoking status**				
Never	804 (60.7%)	240 (52.9%)	38 (62.3%)	1082 (58.8%)
Former	428 (32.3%)	184 (40.5%)	~	631 (34.3%)
Current	73 (5.5%)	21 (4.6%)	~	96 (5.2%)
Missing	~	~	~	30 (1.6%)
**Alcohol consumption status**				
Never	61 (4.6%)	~	~	82 (4.5%)
Former	243 (18.4%)	91 (20.0%)	~	352 (19.1%)
Current	1020 (77.0%)	345 (76.0%)	40 (65.6%)	1405 (76.4%)
**Age at cancer diagnosis**				
<18	~	~	~	~
18-64	1044 (78.9%)	275 (60.6%)	46 (75.4%)	1365 (74.2%)
≥65	268 (20.2%)	174 (38.3%)	~	457 (24.9%)
Missing	~	~	~	~
**Surgery**				
Yes	300 (22.7%)	102 (22.5%)	~	409 (22.2%)
**Chemotherapy**				
Yes	323 (24.4%)	135 (29.7%)	23 (37.7%)	481 (26.2%)
**Hormone Therapy**				
Yes	811 (61.3%)	162 (35.7%)	28 (45.9%)	1001 (54.4%)
**Radiation Therapy**				

© 2023 Shi M et al. *JAMA Network Open.*

Yes	141 (10.6%)	73 (16.1%)	~	224 (12.2%)
**Immunotherapy**				
Yes	180 (13.6%)	102 (22.5%)	~	295 (16.0%)

Abbreviations: EHR, electronic health record; GED, General Educational Development; SD, standard deviation.
a. After restricting to patients with EHR ≥1 year before the baseline survey, we identified 1839 patients with any type of the listed cancer treatment within 1 year of the baseline of survey.
b. Other included participants who selected 'intersex', 'prefer not to answer', 'none of these', and 'skip' when asked 'What was your biological sex assigned at birth?'.
c. Other included individuals reporting races other than Hispanic, non-Hispanic Black or non-Hispanic White and individuals with more than one race/ethnicity.
In accordance with *All of Us* Research Program policy, values corresponding to fewer than 20 participants were removed from the table (labeled as ~).

© 2023 Shi M et al. *JAMA Network Open*.

eTable 4. Adjusted Odds Ratios of Current Drinking Among Cancer Survivors, *All of Us* Research Program

	No. of participants	OR (95% CI)[a]
**Age at survey**		
≥65	6151	Ref
<50	1905	1.14 (1.00, 1.31)
50-64	3759	0.95 (0.86, 1.05)
**Sex**		
Women	7344	Ref
Men	3971	0.99 (0.90, 1.09)
**Race/ethnicity**		
Hispanic	641	0.65 (0.56, 0.76)
Non-Hispanic Black	696	0.71 (0.61, 0.82)
Non-Hispanic White	9378	Ref
Other[b]	466	0.49 (0.41, 0.58)
**Cancer type**		
Non-alcohol-related cancers	7518	Ref
Alcohol-related cancers[c]	4297	1.16 (1.06, 1.27)
**Age at cancer diagnosis**		
≥65	2570	Ref
<18	224	1.33 (0.96, 1.84)
18-64	8955	1.10 (0.98, 1.23)
**Medication and/or receiving treatment[d]**		
No	7547	Ref
Yes	4211	0.87 (0.80, 0.94)
**Smoking status**		
Never	6591	Ref
Former	4262	1.27 (1.16, 1.39)
Current	738	1.44 (1.22, 1.70)

a. Adjusted for age at survey, sex, race/ethnicity, marital status, education level, annual household income, insurance status, smoking status, cancer type, age at cancer diagnosis, and currently prescribed medication and/or receiving treatment.

b. Other included individuals reporting races other than Hispanic, non-Hispanic Black or non-Hispanic White and individuals with more than one race/ethnicity.

c. Alcohol-related cancers included breast, colon & rectum, and head & neck cancer. Esophageal cancer was not included since the association with alcohol drinking is largely confined to squamous cell carcinoma whereas the majority esophageal cancer were adenocarcinoma in the US. Liver cancer was not included as it was not specifically asked in the *All of Us* Research Program survey.

d. Self-reported current medication prescription and/or treatment in the *Personal Medical History* survey.

© 2023 Shi M et al. *JAMA Network Open*.

eTable 5. Adjusted Odds Ratios of Risky Drinking Behaviors Among Current Drinking Cancer Survivors, *All of Us* Research Program

	Exceeding Moderate Drinking[a]		Binge Drinking[b]		Hazardous Drinking[c]	
	No. of participants	OR (95% CI)[d]	No. of participants	OR (95% CI)[d]	No. of participants	OR (95% CI)[d]
**Age at survey**						
≥65	582	Ref	966	Ref	2365	Ref
<50	410	2.90 (2.41, 3.48)	817	4.46 (3.85, 5.15)	776	1.12 (0.98, 1.27)
50-64	549	1.84 (1.58, 2.15)	1029	2.15 (1.90, 2.43)	1386	0.93 (0.84, 1.03)
**Sex**						
Women	777	Ref	1560	Ref	2946	Ref
Men	696	2.38 (2.09, 2.72)	1119	2.10 (1.89, 2.34)	1581	0.90 (0.82, 0.98)
**Race/ethnicity**						
Hispanic	128	1.31 (1.04, 1.64)	233	1.31 (1.09, 1.58)	212	0.84 (0.70, 1.00)
Non-Hispanic Black	120	1.04 (0.83, 1.31)	233	1.38 (1.15, 1.66)	200	0.68 (0.57, 0.82)
Non-Hispanic White	1155	Ref	2071	Ref	3888	Ref
Other[e]	57	0.87 (0.64, 1.18)	114	0.94 (0.74, 1.18)	147	0.62 (0.51, 0.76)
**Cancer type**						
Non-alcohol-related cancers	1110	Ref	1966	Ref	2849	Ref
Alcohol-related cancers[f]	431	0.96 (0.84, 1.10)	846	0.96 (0.86, 1.07)	1678	1.04 (0.95, 1.14)
**Age at cancer diagnosis**						
≥65	255	Ref	394	Ref	987	Ref
<18	56	1.52 (1.04, 2.24)	106	1.71 (1.24, 2.35)	98	1.52 (1.11, 2.08)
18-64	1221	1.07 (0.89, 1.27)	2297	1.20 (1.04, 1.39)	3414	0.99 (0.88, 1.10)
**Medication and/or receiving treatment[g]**						
No	972	Ref	1784	Ref	2929	Ref
Yes	563	1.04 (0.93, 1.17)	1017	0.96 (0.86, 1.07)	1582	0.96 (0.88, 1.04)
**Smoking status**						
Never	560	Ref	1325	Ref	2208	Ref
Former	701	2.46 (2.16, 2.79)	1107	1.69 (1.53, 1.87)	1925	1.83 (1.68, 1.99)
Current	248	4.14 (3.40, 5.04)	329	2.27 (1.91, 2.71)	319	2.13 (1.79, 2.53)

Abbreviations: AUDIT-C, Alcohol Use Disorders Identification Test-Consumption; CI, confidence interval; OR, odds ratio.
a. Defined as >2 drinks on a typical day when they drink in the past year.
b. Defined as having ≥6 drinks on one occasion in the past year.
c. Defined as AUDIT-C score ≥3 for women and ≥4 for men in the past year.
d. Adjusted for age at survey, sex, race/ethnicity, marital status, education level, annual household income, insurance status, smoking status, cancer type, age at cancer diagnosis, and currently prescribed medication and/or receiving treatment.
e. Other included individuals reporting races other than Hispanic, non-Hispanic Black or non-Hispanic White and individuals with more than one race/ethnicity.
f. Alcohol-related cancers included breast, colon & rectum, and head & neck cancer. Esophageal cancer was not included since the association with alcohol drinking is largely confined to squamous cell carcinoma whereas the majority esophageal cancer were adenocarcinoma in the US. Liver cancer was not included as it was not specifically asked in the *All of Us* Research Program survey
g. Self-reported current medication prescription and/or treatment in the *Personal Medical History* survey.

© 2023 Shi M et al. *JAMA Network Open.*

eTable 6. Prevalence of Alcohol Consumption Patterns Among Survey Participants Without Prior Cancer Diagnosis According to Sex, *All of Us* Research Program

Characteristics	Women	Men	Other[a]	Total
**Alcohol consumption status**				
No. of participants	78518	40696	763	119977
Never	5013 (6.4%)	1898 (4.7%)	73 (9.6%)	6984 (5.8%)
Former	10805 (13.8%)	5992 (14.7%)	138 (18.1%)	16935 (14.1%)
Current	62700 (79.9%)	32806 (80.6%)	552 (72.3%)	96058 (80.1%)
**Risky drinking behaviors among current drinkers**				
**Exceeding moderate drinking**[b]	11366 (18.1%)	8459 (25.8%)	124 (22.5%)	19949 (20.8%)
**Binge drinking**[c]	20568 (32.8%)	13420 (40.9%)	147 (26.6%)	34135 (35.5%)
<1 per month	15845 (25.3%)	8898 (27.1%)	97 (17.6%)	24840 (25.9%)
≥1 per month	4723 (7.5%)	4522 (13.8%)	50 (9.0%)	9295 (9.7%)
**Hazardous drinking**				
AUDIT-C score, mean (SD)[d]	2.70 (1.69)	3.40 (2.04)	2.86 (1.85)	2.94 (1.85)
Hazardous drinking[e]	28180 (44.9%)	19661 (59.9%)	249 (45.1%)	48090 (50.1%)

Abbreviations: AUDIT-C, Alcohol Use Disorders Identification Test-Consumption; SD, standard deviation.
a. Other included participants who selected 'intersex', 'prefer not to answer', 'none of these', and 'skip' when asked 'What was your biological sex assigned at birth?'.
b. Defined as >2 drinks on a typical day when they drink in the past year.
c. Defined as having ≥6 drinks on one occasion in the past year.
d. Calculated by adding scores of three questions in the AUDIT-C questionnaire.
e. Defined as AUDIT-C score ≥3 for women and ≥4 for men in the past year.
In accordance with *All of Us* Research Program policy, values corresponding to fewer than 20 participants were removed from the table (labeled as ~).

© 2023 Shi M et al. *JAMA Network Open.*

eFigure 1. Flow Chart of the Study Population

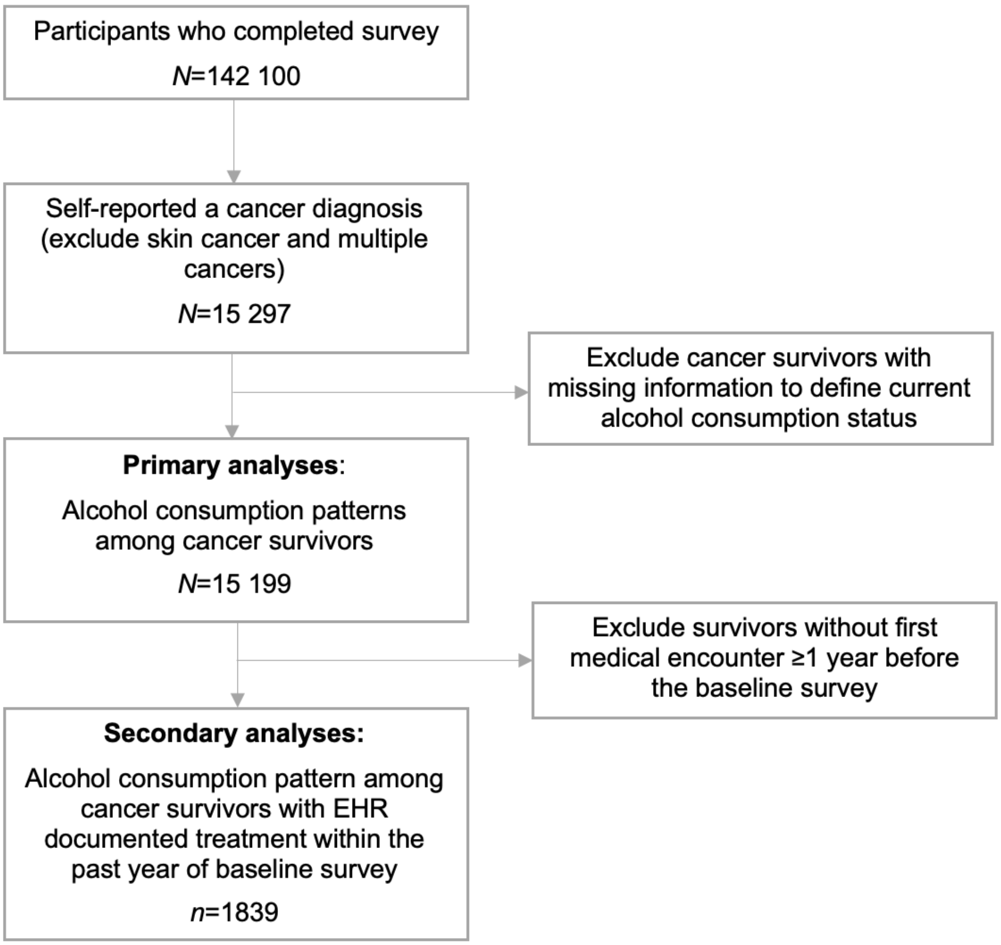

© 2023 Shi M et al. *JAMA Network Open*.

eFigure 2. (A) Mean AUDIT-C Score[a] Among Cancer Survivors According to Sex; (B) Venn Diagram Showing Cancer Survivors Engaged in Exceeding Moderate Drinking, Binge Drinking and Hazardous Drinking Among 11815 Current Drinkers, *All of Us* Research Program

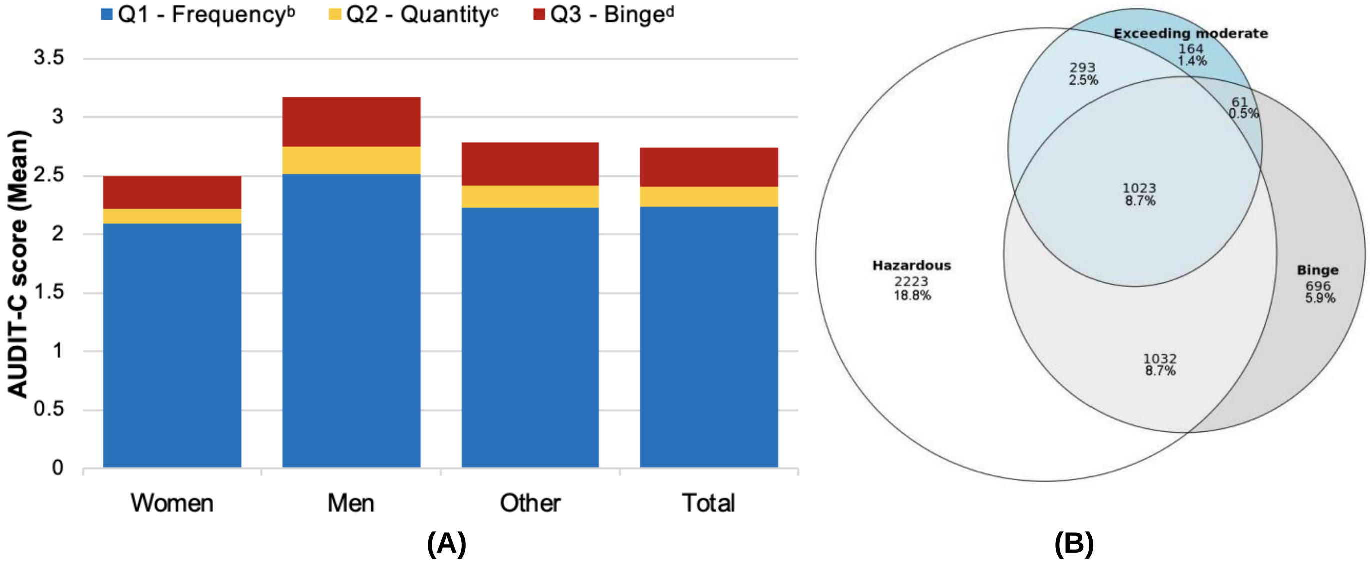

Abbreviation: AUDIT-C, Alcohol Use Disorders Identification Test-Consumption.
a. Calculated by adding scores of three questions in the AUDIT-C questionnaire (0-12 points).
b. “How often did you have a drink containing alcohol in the past year?” with options of never (0 point), monthly or less (1 point), two or four times a month (2 points), two to three times a week (3 points), or four or more times a week (4 points).
c. “On a typical day when you drink, how many drinks do you have?” with options of 1 or 2 (0 point), 3 or 4 (1 point), 5 or 6 (2 points), 7 to 9 (3 points), or 10 or more (4 points).
d. “How often did you have six or more drinks on one occasion in the past year?” with options of never (0 point), less than monthly (1 point), monthly (2 points), weekly (3 points), or daily or almost daily (4 points).

© 2023 Shi M et al. *JAMA Network Open*.

eFigure 3. Mean AUDIT-C Score[a] Among Cancer Survivors According to Age at Cancer Diagnosis and Smoking Status, *All of Us* Research Program

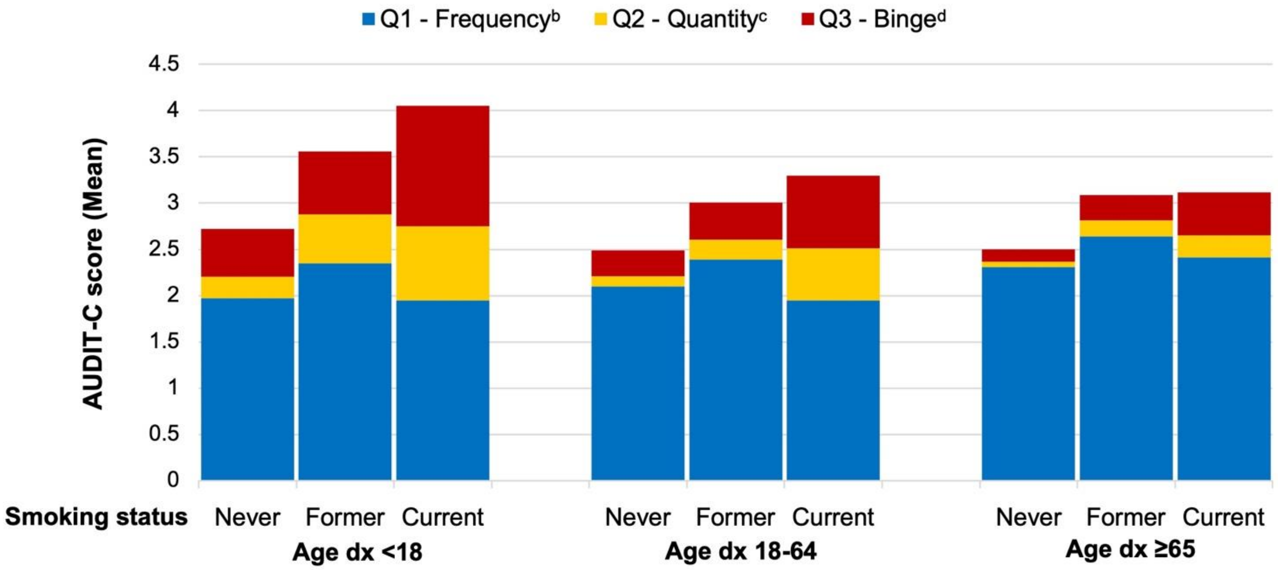

Abbreviation: AUDIT-C, Alcohol Use Disorders Identification Test-Consumption; dx, diagnosis.
a. Calculated by adding scores of three questions in the AUDIT-C questionnaire (0-12 points).
b. "How often did you have a drink containing alcohol in the past year?" with options of never (0 point), monthly or less (1 point), two or four times a month (2 points), two to three times a week (3 points), or four or more times a week (4 points).
c. "On a typical day when you drink, how many drinks do you have?" with options of 1 or 2 (0 point), 3 or 4 (1 point), 5 or 6 (2 points), 7 to 9 (3 points), or 10 or more (4 points).
d. "How often did you have six or more drinks on one occasion in the past year?" with options of never (0 point), less than monthly (1 point), monthly (2 points), weekly (3 points), or daily or almost daily (4 points).

© 2023 Shi M et al. *JAMA Network Open*.
