# Supplementary material for: Alcohol Consumption Among Adults With a Cancer Diagnosis in the All of Us Research Program
Source: JAMA Netw Open. 2023 Aug 10;6(8):e2328328. doi: 10.1001/jamanetworkopen.2023.28328 (PMC10415957; doi:10.1001/jamanetworkopen.2023.28328)
Supplement: Supplement 2. — Data Sharing Statement [file jamanetwopen-e2328328-s002.pdf]

Data Sharing Statement

Shi. Alcohol Consumption Among Adults With a Cancer Diagnosis in the All of Us Research Program. *JAMA Netw Open*. Published August 10, 2023.
doi:10.1001/jamanetworkopen.2023.28328

Data
Data available: No

Additional Information
Explanation for why data not available: Data can be accessed via the All of Us Research Workbench (researchallofus.org).
